# Impedance, Electrical Equivalent Circuit (EEC) Modeling, Structural (FTIR and XRD), Dielectric, and Electric Modulus Study of MC-Based Ion-Conducting Solid Polymer Electrolytes

**DOI:** 10.3390/ma15010170

**Published:** 2021-12-27

**Authors:** Balen K. Faris, Ary A. Hassan, Shujahadeen B. Aziz, Mohamad A. Brza, Aziz M. Abdullah, Ari A. Abdalrahman, Ola A. Abu Ali, Dalia I. Saleh

**Affiliations:** 1 Hameed Majid Advanced Polymeric Materials Research Lab., Physics Department, College of Science, University of Sulaimani, Qlyasan Street, Kurdistan Regional Government, Sulaimani 46001, Iraq; balen.07001510@univsul.edu.iq (B.K.F.); ary.07001490@univsul.edu.iq (A.A.H.); ari.abdulla@univsul.edu.iq (A.A.A.); 2 Department of Civil Engineering, College of Engineering, Komar University of Science and Technology, Kurdistan Regional Government, Sulaimani 46001, Iraq; 3 Medical Physics Department, College of Medicals & Applied Science, Charmo University, Chamchamal 46023, Sulaimani, Iraq; mohamad.brza@gmail.com (M.A.B.); aziz.abdullah@charmouniversity.org (A.M.A.); 4 Department of Chemistry, College of Science, Taif University, P.O. Box 11099, Taif 21944, Saudi Arabia; o.abuali@tu.edu.sa (O.A.A.A.); daliarawan@yahoo.com (D.I.S.)

**Keywords:** methylcellulose polymer electrolyte, XRD and FTIR, impedance study, EEC modeling, dielectric study, electric modulus analysis, relaxation processes

## Abstract

The polymer electrolyte system of methylcellulose (MC) doped with various sodium bromide (NaBr) salt concentrations is prepared in this study using the solution cast technique. FTIR and XRD were used to identify the structural changes in solid films. Sharp crystalline peaks appeared at the XRD pattern at 40 and 50 wt.% of NaBr salt. The electrical impedance spectroscopy (EIS) study illustrates that the loading of NaBr increases the electrolyte conductivity at room temperature. The DC conductivity of 6.71 × 10^−6^ S/cm is obtained for the highest conducting electrolyte. The EIS data are fitted with the electrical equivalent circuit (EEC) to determine the impedance parameters of each film. The EEC modeling helps determine the circuit elements, which is decisive from the engineering perspective. The DC conductivity tendency is further established by dielectric analysis. The EIS spectra analysis shows a decrease in bulk resistance, demonstrating free ion carriers and conductivity boost. The dielectric property and relaxation time confirmed the non-Debye behavior of the electrolyte system. An incomplete semicircle further confirms this behavior model in the Argand plot. The distribution of relaxation times is related to the presence of conducting ions in an amorphous structure. Dielectric properties are improved with the addition of NaBr salt. A high value of a dielectric constant is seen at the low frequency region.

## 1. Introduction

The focus on polymers has been further increased with technology and science. However, more limitations have arisen with the enlargement of the latest class of polymers, such as the supply of raw material, synthesis technology, and production cost [1]. Polymer electrolytes (PEs) are macromolecular systems with which ions and protons can transport charge. The majority of typical applications of PEs are in energy conversion devices and storage devices such as supercapacitors (SCs), fuel cells, and batteries [2,3,4,5,6]. These materials were first mentioned by Fenton et al. in 1973 [2]. PEs consist of dissolved salts in polymer matrices with high molecular mass [7,8,9]. These materials are better than the traditional liquid electrolytes. For example, an ionic-conducting phase has a transport property compared with other liquid ionic solutions, in addition to being lightweight, having relatively high ionic conductivity, solvent-free, flexibility, and thin film forming capability. In addition, PEs are safe and free from the problem of electrolyte leakage [7,9,10]. The first ion-conducting polymer was poly(ethylene oxide) (PEO) that incorporated with alkali metal salts in 1973 [11]. A DC conductivity of 5.817 × 10^−7^ S/cm was obtained with 30 wt.% ammonium tetrafluoroborate (NH_4_BF_4_) in the chitosan:PEO (CS:PEO) polymer blend [12]. Adding 30 wt.% NH_4_BF_4_ into a CS:potato starch (PS) provided the conductivity 3.07 × 10^−8^ S/cm [13]. Salehan et al. [14] reported that the loading of 2 wt.% aluminium oxide (Al_2_O_3_) into a corn starch (CS):lithium iodide (LiI) provided a conductivity of (6.73 ± 0.78) × 10^–4^ S/cm. Shetty et al. [15] documented that the loading of 20 wt.% sodium bromide (NaBr) into carboxymethyl cellulose (CMC) provided a conductivity of ~5.15 × 10^–4^ S/cm.

From the necessary viewpoint, ionic conduction in solid polymer electrolytes (SPEs) is not well understood. This is because the property of ion transport relies on many factors, including salt concentration, degree of salt dissociation, degree of ion aggregation, the dielectric constant of host polymer, and polymer chains mobility [8,10,16,17,18,19].

The polymer electrolyte dielectric analysis delivers additional information even though these materials possess an elevated conductivity value. Thus, the phenomena of dielectric relaxation study are an influential tool for comprehending ion transport behavior and gaining information of molecular/ionic interaction in polymer electrolytes [20].

Dielectric measurements, for example dielectric loss and constant, provide valuable insight into polymers’ physical and chemical properties. These properties can be dramatically prejudiced by incorporating other polymers or a filler to the polymer [21,22]. More studies were performed on Li salt interacted PE, but fewer efforts were made on PE-based sodium interacted films. Using sodium in PE has more advantages over its lithium counterpart. Sodium is more abundant and cheaper than lithium [23]. Hence, the sodium-ion battery has drawn more attention as the next generation secondary battery as it is 40% cheaper than a lithium-ion battery. Fuentes et al. [24] documented that lithium salt PEs are the most suitable and, therefore, the most commonly used in the new battery generation design. However, their study found that Na^+^ ions produce a higher ionic conductivity or at least a similar conductivity to Li^+^ ions in the same primary material. This provided an outstanding opportunity to use Na^+^ ions rather than lithium in constructing a new battery generation, improving conductivity, and ensuring widespread supply. NaBr has a small lattice energy of 747 kJ/mol, which is marginally lower than NaCl (786 kJ/mol) as the size of Cl^−^ (167 pm) is smaller than the size of Br^−^ (182 pm) [12,25]. The salts lattice energy, such as sodium salts used in PEs, can considerably impact the conductivity of the synthesized electrolyte. Two factors determine the salts lattice energy. The first is the charge of the ions and the second is their size. The charge ion raises the lattice energy of the salt, whereas the ion size decreases the salt lattice energy.

Railanmaa et al. [26] presented gelatin gel electrolyte properties in the printed supercapacitor, and they studied the mechanical and electrical behaviors of the devices. The authors focused on gel performance. The gel electrolyte performance was similar to an aqueous liquid electrolyte (LE) with a similar salt as the ionic conductor with respect to the main features: equivalent series resistance, capacitance, and leakage current. With a 2 M NaCl gel electrolyte, the performance of electrical equivalent to that of a 1 M LE was obtained, and the devices endured bending down to a 10 mm radius [26].

In this research, the effect of NaBr salt on the dielectric property of MC-based electrolytes was studied. The structural behaviors of the films were examined. The relaxation process was analyzed in more detail with respect to electric modulus and Tanδ spectra. From the viewpoint of engineering, materials with a small value of Tanδ are more vital for electronics and devices application.

## 2. Experimental Details

### 2.1. Raw Materials and Sample Preparation

Both MC (4000 cp) and NaBr(102.894 g/mol) were supplied by Sigma-Aldrich and used in this research. The solid polymer preparations were carried out in aqueous solution using distilled water. It comprised dissolution of 1 g of MC powder in 100 mL of 1% acetic acid by stirring using a magnetic stirrer for around 24 h at room temperature. The stirring continued until homogeneous clear and viscose solutions were obtained. In the preparation of MC/NaBr SPE system, different quantities of NaBr (10, 20, 30, 40, and 50 wt.%) were loaded into a series of solutions of solid polymers with continuous stirring. This series of solutions was cast into Petri dishes to dry at room temperature for films to form. The films were transferred into a desiccator with silica gel desiccants to keep dry. Table 1 summarizes the main components of the prepared samples.

### 2.2. Electrochemical Impedance Spectroscopy (EIS) Technique

At ambient temperature, the EIS of the prepared films was acquired using an HIOKI 3531 Z LCR Hi-tester within a frequency ranged between 50 and 5000 kHz. The LCR meter was hyphenated to a computer where the imaginary and real parts of the EIS were displayed. The films were sandwiched between 2 identical circular stainless steel electrodes in the form of discs with a 2 cm diameter and sandwiched under spring pressure to ensure the desired contact.

## 3. Results and Discussion

### 3.1. FTIR and XRD Analysis

**Figure 1:**
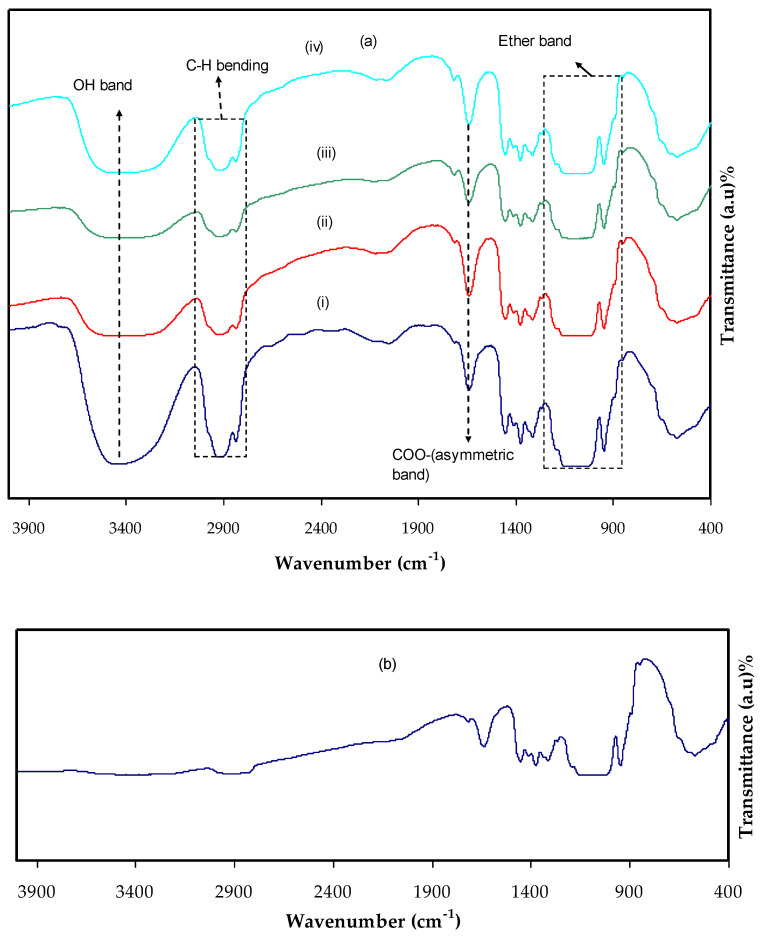
FTIR spectra at a wave number between 400 cm^−1^ to 4000 cm^−1^ (**a**) for (i) MCNAB1, (ii) MCNAB2, (iii) MCNAB3, and (iv) MCNAB4, and (**b**) for MCNAB5 electrolyte films.

Figure 1a,b shows the FTIR spectra of each film. Hydrogen bond creation in MC electrolyte is explained by IR spectroscopy as hydrogen bonding changes the stretching vibration frequency [27,28]. The hydroxyl band in pure MC and MCNAB electrolyte films is related to the wavenumber ranged from 3435 to 3447 cm^−1^, while the ether band emerged at 1047 and 1113 cm^−1^ [29], and the absorption bands shifted and intensity decreased in the electrolyte films. The absorption bands at 1419, 1609, 2927, and 3418 cm^−1^ in the MCNAB electrolyte films are related to the symmetrical stretching vibration of COO-, asymmetrical stretching vibration of COO-, aliphatic C-H, and O-H, respectively [30]; however, the intensity of the peaks decreased and shifted from MCNAB1 to MCNAB5. The peaks related to C-H stretching vibration at 2901 cm^−1^, O-H stretching vibration at 3447 cm^−1^, C-O stretching vibration from asymmetric oxygen bridge between 1057 and 1113 cm^−1^, C-O carbonyl stretching vibration from the glucose of the cellulose at 1635 cm^−1^, and O-CH_3_ stretching vibration at 938 cm^−1^ are seen in pure MC [27]. In the case of MCNAB electrolyte films, these peaks shift and intensity decreases, while addition of 50 wt.% NaBr causes it to decrease noticeably as revealed in Figure 1b. Small absorption peaks between 1247 and 1448 cm^−1^ are connected to C-H bending vibration of MC. Small bands between 478 and 648 cm^−1^ are related to C-H vibration [31]. These bands shifted in their position and decreased in intensity in the case of the MCNAB electrolyte films. The free or unbounded hydroxyl group are related to 3649 to 3511 cm^−1^. The hydrogen-bound hydroxyl group existence caused a shift of absorption to lower frequency while the intensity was increased and the band widened, and an asymmetrical peak emerged. The hydroxyl absorption measures the interaction of hydrogen bonding and the hydrogen bond strength in the polymer. The MC spectrum region that is relevant is between 3799 and 3001 cm^−1^, and the spectrum was inferred in terms of hydroxyl stretching at 3459 cm^−1^ [28]. The 950–1250 cm^−1^ region is more informative from the perspective of the issues solved herein. The 947–1249 cm^−1^ range includes a complex robust absorption band of MC which is owing to C-O bond stretching vibration in the spectra of cellulose and its ether [32].

**Figure 2:**
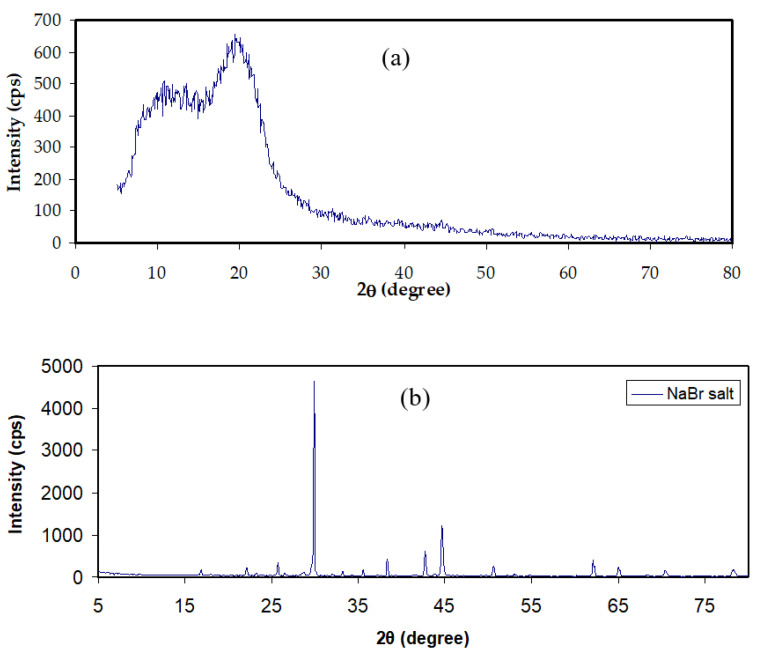
XRD pattern of (**a**) neat MC film and (**b**) NaBr salt.

**Figure 3:**
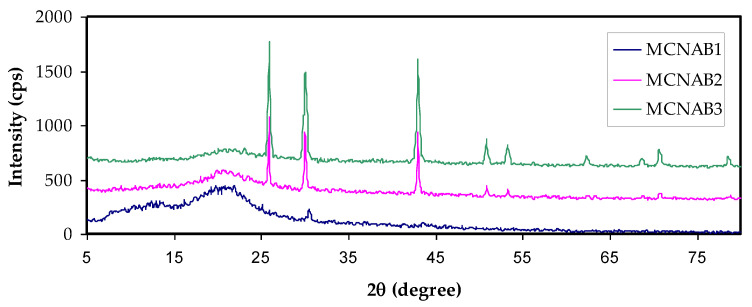

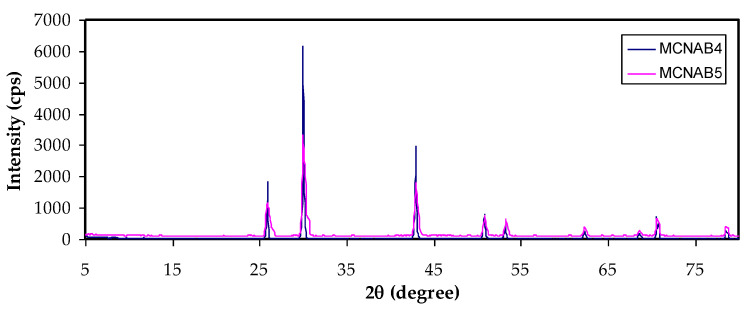
XRD pattern of MC:NaBr solid electrolyte films.

Figure 2a,b shows the XRD pattern of MC and NaBr salt, and MC-doped samples are shown in Figure 3. It was mentioned that the wide crystalline hump that appeared at around 2θ between 19° and 21° relates to the intermolecular hydrogen bonding and shows a short distance order of the film chains in MC [33,34]. It is seen that for 20 and 30 wt.% of NaBr, the intensity decreased, the broadening increased, and some new crystalline peaks emerged. This broadening is due to the distraction of the crystalline nature of the polymer by loading NaBr [34]. The XRD analysis revealed an increase in the amorphous structure in the MC:NaBr films, whereas the MCNAB4 and MCNAB54 samples’ spectra revealed several high-intensity peaks related to the undissolved salts in these samples and their leakage through the film surface. As displayed in Figure 2b and Figure 3, the XRD peaks of the undissolved salt appeared in the electrolyte films. The XRD results are in agreement with EIS results in the next section as the conductivity decreased for the MCNAB4 and MCNAB54 samples.

### 3.2. Study of Impedance Plots

**Figure 4:**
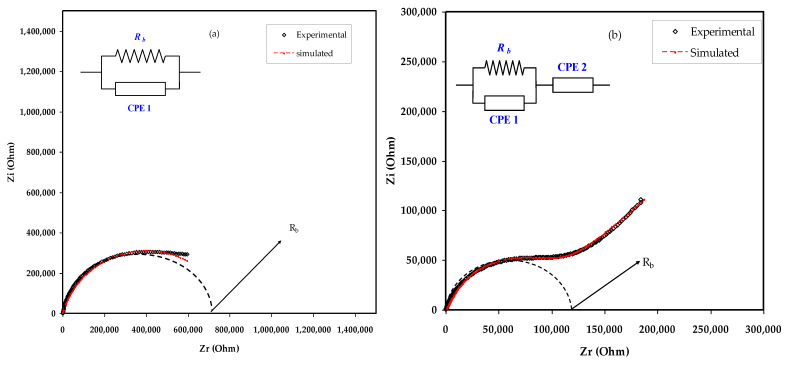

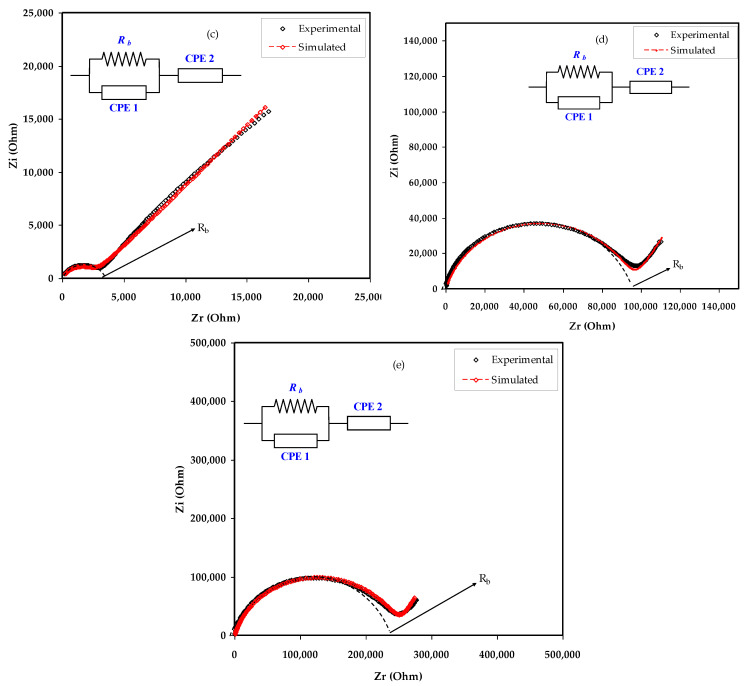
Experimental and fitting (EEC) impedance (Nyquist) plots for (**a**) MCNAB 1, (**b**) MCNAB 2, (**c**) MCNAB 3, (**d**) MCNAB 4, and (**e**) MCNAB 5 samples.

Indispensable marks which differentiate PEs from ionic conductors obtained by dissolving salts in small molar mass solvents begin from the cation motion mechanism. The electrical impedance responses, including the Z′ vs. Z″, for the MC:NaBr electrolyte films are displayed in Figure 4a–e. The EEC as a modeling is regularly used in the impedance spectroscopy analysis, because it is straightforward and explains a broad picture [35]. The experimental impedance responses can be explained with respect to the EEC that comprises bulk resistance (*R_b_*) and the constant phase elements of CPE1 and CPE2 for the charge carriers within the sample as exhibited in insets of Figure 4.

The elevated frequency section is the location of *R_b_* and CPE1 responses, and at the low frequency area, CPE2 appears. The CPE2 response results from the double layer capacitance between the electrodes and polymer electrolyte at the low frequency region. It is well known that in a definite system, the CPE term is further often used in equivalent circuits in place of the perfect capacitor. This nomination is owed because, in SPE, there is pseudocapacitor behavior dissimilar from that of a pure or idyllic capacitor in an ideal semicircular model [36].

Figure 4a–e exhibits the impedance responses and corresponding ECs for all blend samples. In Figure 4a, a complex impedance response is shown that consists of a depressing semicircle at the region of high frequency. The equivalent circuit is expressed as a parallel arrangement of *R_b_* and CPE in Figure 4a. Based on experimental results, the equivalent circuit is expressed as a parallel combination of *R_b_* and bulk capacitance (CPE) in series with another CPE originating from the tilted point area as presented in Figure 4b–e. The mathematical basis of impedance of *Z_CPE_* is shown below [37,38,39]:

(1) $$Z_{CPE}=\frac{\cos(\pi n/2)}{Y_{m}\omega^{n}}-j\frac{\sin(\pi n/2)}{Y_{m}\omega^{n}}$$

where *Y_m_* is the CPE capacitance, angular frequency is referred by ω, and *n* is the measure of extent of the deviation of the plot from the imaginary axis. Herein, the *Z_r_* and *Z_i_* of complex impedance (Z*) in the corresponding circuit (inset of Figure 4a) is shown below:

(2) $$Z_{r}=\frac{R_{1}+R_{1}^{2}Y_{1}\omega^{n1}\cos(\pi n_{1}/2)}{1+2R_{1}Y_{1}\omega^{n_{1}}\cos(\pi n_{1}/2)+R_{1}^{2}Y_{1}^{2}\omega^{2n_{1}}}$$

(3) $$Z_{i}=\frac{R_{1}^{2}Y_{1}\omega^{n_{1}}\sin(\pi n_{1}/2)}{1+2R_{1}Y_{1}\omega^{n_{1}}\cos(\pi n_{1}/2)+R_{1}^{2}Y_{1}^{2}\omega^{2n_{1}}}$$

Herein, the *Z_r_* and *Z_i_* of Z* in the EC (inset of Figure 4b–e) is shown below:

(4) $$Z_{r}=\frac{R_{1}+R_{1}^{2}Y_{1}\omega^{n1}\cos(\pi n_{1}/2)}{1+2R_{1}Y_{1}\omega^{n_{1}}\cos(\pi n_{1}/2)+R_{1}^{2}Y_{1}^{2}\omega^{2n_{1}}}+\frac{\cos(\pi n_{2}/2)}{Y_{2}\omega^{n_{2}}}$$

(5) $$Z_{i}=\frac{R_{1}^{2}Y_{1}\omega^{n_{1}}\sin(\pi n_{1}/2)}{1+2R_{1}Y_{1}\omega^{n_{1}}\cos(\pi n_{1}/2)+R_{1}^{2}Y_{1}^{2}\omega^{2n_{1}}}+\frac{\sin(\pi n_{2}/2)}{Y_{2}\omega^{n_{2}}}$$

Table 2 presents all the parameters extracted from the fitting of the impedance plots using ECs. The *R_b_* in the equivalent circuit represents the bulk resistance, as presented in the inset of Figure 4. The *R_b_* value can be extracted from the figure by intersecting the semicircle with the *Z_r_* axis of the spectrum, as presented in Table 2. Given the *R_b_* and the film thickness, one can measure the DC conductivity using Equation (6) [13,40],

(6) $$\sigma_{dc}=\left(\frac{1}{R_{b}}\right)\times \left(\frac{t}{A}\right)$$

where *t* stands for the film thickness and the area of the electrodes is represented by *A*. The DC conductivity and *R_b_* values of the films are presented in Table 2.

Li et al. [41] prepared an organic-inorganic reticular PE. Isocyanate connects PEO molecular chains and fumed silica. The PEO-TDI-SiO_2_SPEs developed can considerably have increased conductivity of 0.12 mS cm^−1^ at room temperature. This is due to the fact that the TDI-SiO_2_ nanoparticles prevent crystallization of the polymer that offers more Li-ion transport pathways.

### 3.3. Dielectric and Electric Modulus Analysis

**Figure 5:**
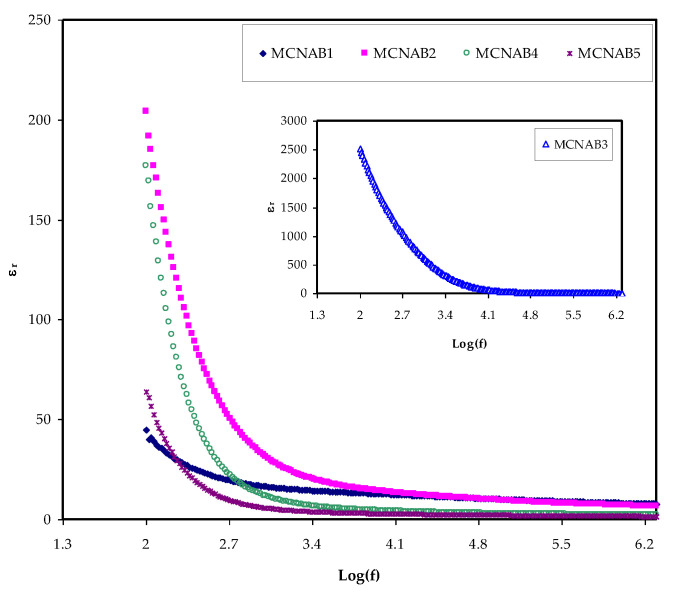
*ε_r_* against frequency at room temperature for each sample.

**Figure 6:**
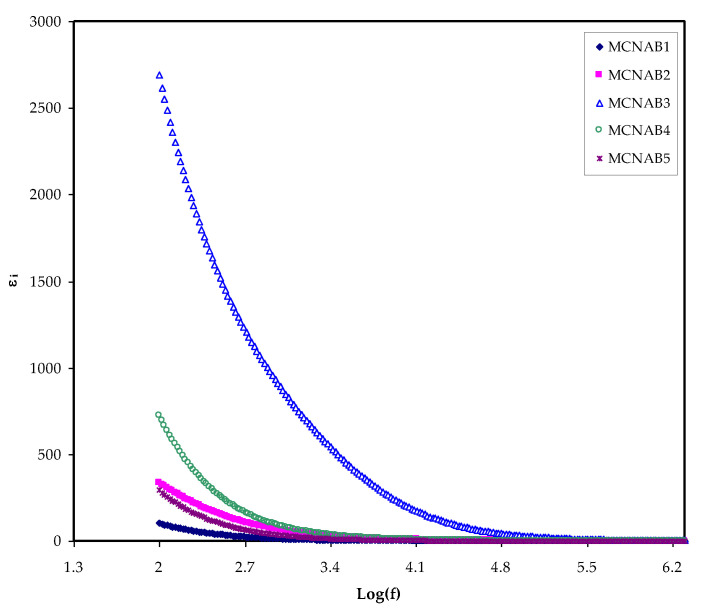
*ε_i_* against frequency at room temperature for each sample.

The most effective method for determining the increase in the free ions density in the PEs, which results in increased conductivity, is to determine the dielectric property [42]. The ion transport process relies powerfully on a few factors; the salt dissociation degree and its concentration, the dielectric constant (*ɛ_r_*) of polymer, the extent of ion agglomeration, and polymer chains mobility. To better understand conductivity processes, it is necessary to consider the dielectric properties of ionically conducting polymer electrolytes [20]. A unique aspect of PEs is that ion journey occurs lacking long-range displacement of the solvent. Despite serious research, the method of ionic conductivity in polymer electrolytes is still not extensively discussed. Ion transport in PEs is an intricate process counting ion movement, local movement of the polymer segment, and intra- and inter polymer transport among ion coordination sites. One extra vital note is that the *ε_r_* and *ε_i_* parts increase with decreasing frequency. The *ε_r_* and *ε_i_* are revealed in Figure 5 and Figure 6. The relatively high values of *ε_r_* and *ε_i_* at the stumpy frequency region are due to the contribution of electrode polarization at the interfacial region to the impedance of the samples [43,44]. On the one hand, a high involvement of charge accretion at the electrodes–electrolytes interfaces occurs at the low frequency. On the other hand, the periodic setbacks of the electric field (EF) happen rapidly where there is no overload of diffusion of ion in the field direction at the high frequencies. Therefore, the polarization from charge build-up diminishes, resulting in a lesson in both the *ε_r_* and *ε_i_* as shown in Figure 5 and Figure 6 [43].

In the study of dielectric relaxation, one can understand the performance of PEs. Therefore, it is helpful to use a broad frequency range during dielectric relaxation measurements to deal with dipole relaxation in polymeric materials [37]. From the *Z_r_* and *Z_i_* parts of Z*, and the ones of complex electric modulus (M*) and complex permittivity (ε*) can be calculated, using the following relationships [17,18,37,45,46,47],

(7) $$\varepsilon_{r}=\frac{Z_{i}}{\omega C_{o}\left(Z_{r}^{2}+Z_{i}^{2}\right)}$$

(8) $$\varepsilon_{i}=\frac{Z_{r}}{\omega C_{o}\left(Z_{r}^{2}+Z_{i}^{2}\right)}$$

(9) $$M_{r}=Z_{i}C_{o}\omega$$

(10) $$M_{i}=Z_{r}C_{o}\omega$$

where the real part and imaginary part of M* are symbolized as *M*_r_ and *M*_i_ and the dielectric constant and dielectric loss are symbolized as *ε_r_* and *ε_i_*, correspondingly. Here, *C_o_* refers to the vacuum capacitance under study, which is obtained from *ε_o_* A/t (where *A* and *t* are the area and thickness of each film, respectively), and ω is the angular frequency which is equal to 2π*f*, where *f* is the frequency in Hz.

**Figure 7:**
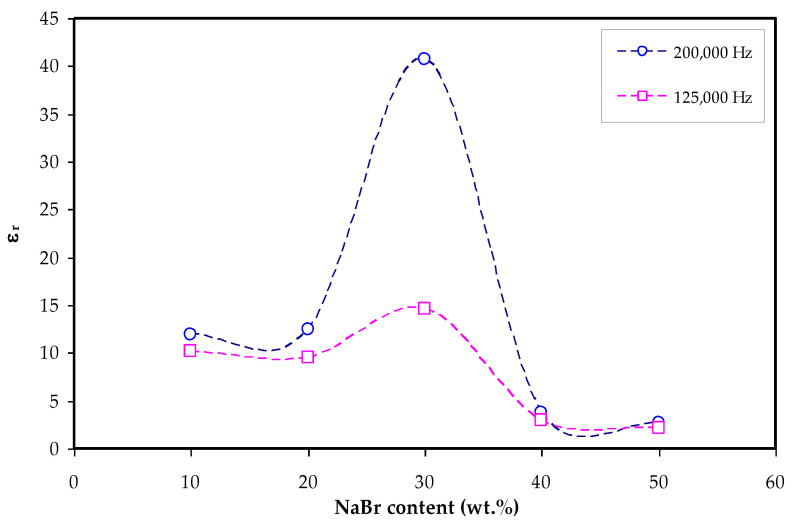
*ε_r_* (at 200,000 and 125,000 Hz) vs. NaBr concentration.

It is seen in Figure 7 with the loading of 30 wt.% NaBr into the MC polymer, the dielectric constant has the highest value due to more free ions and this modification is also seen in the inset of Figure 5. The dielectric constant is decreased at 40 wt.% and 50 wt.% NaBr due to ion association as shown in Figure 7. The *ε_r_* and *ε_i_* values are determined using Equations (7) and (8).

Furthermore, the dielectric loss tangent (*tanδ*) was obtained for each electrolyte to comprehend the electrolytes relaxation mechanisms. The *tanδ* is a ratio between energy dispersed and energy stored in an EF called the dissipation factor [48]. The *tanδ* is obtained using Equation (11) [48].

(11) $$tan\delta =\frac{\varepsilon_{i}}{\varepsilon_{r}}$$

**Figure 8:**
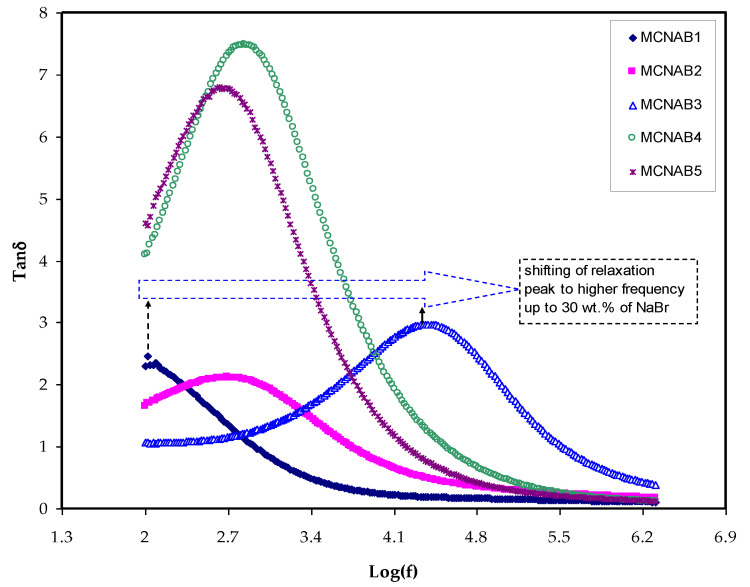
The tan *δ* for the samples.

The relaxation mechanisms of PEs are specifically investigated using loss tangent peaks. The polymer electrolytes dipoles are explained on the basis of dielectric relaxation [37,49]. Figure 8 indicates the loss *tanδ* dielectric relaxation versus frequency for all films. In Figure 8, the peak of the tangent delta is shifted to the high frequency side of the loss tangent peak, indicating that the relaxation process does not occur in the electrolyte. It is a well-known fact that the permanent dipole or induced dipoles are responsible for conductivity and dielectric relaxation peak to appear in the graph [37,49]. The peaks in Figure 8 illustrate the ion dynamics translation associated with the conductivity relaxation of ions. The tan *δ* increased when the frequency is increased as a result of the active element (ohmic) dominant in comparison with the capacitive reactive element. Following that, the *tanδ* achieves a maximum and then decreases at a high frequency due to the active element independence and the reactive element’s dominance [50]. The electrolyte relaxation process presented by the tan *δ* plot indicates the non-Debye behavior of the sample [51].

**Figure 9:**
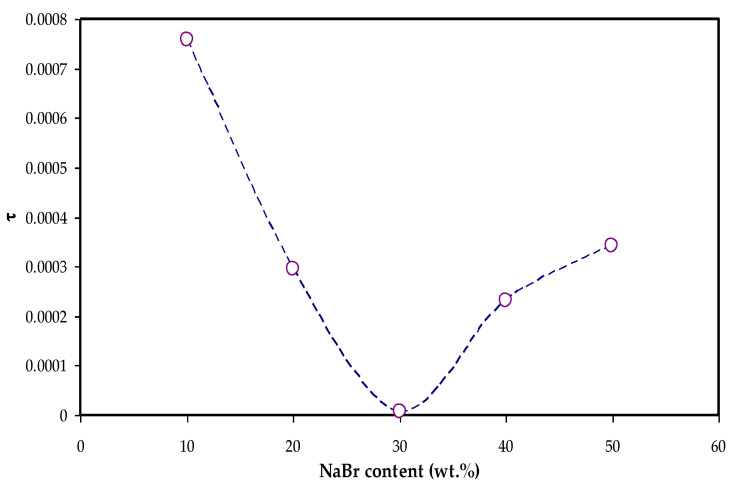
Relaxation time vs. NaBr concentration.

Moreover, the *tanδ* value located at the maximum frequency is denoted as the tan *δ* maximum (*tanδ_max_*) value that is used to determine the relaxation peak angular frequency (*ω_peak_*). Thus, the electrolytes’ relaxation time (τ) is calculated by the reciprocal of *ω_peak_* (1/*ω_peak_*). The measured τ values against NaBr concentrations are shown in Figure 9.

The relaxation time was observed to decrease as the conductivity increased as given in Table 2 and Figure 9. It is seen in Figure 9 that the relaxation time is increased at 40 wt.% and 50 wt.% NaBr, and the conductivity is decreased due to ion association, as seen in Table 2. This is because the ions are mostly connected to the polymer chains during the polymer segments motion, which facilitates hopping between the conduction sites [52]. The smaller relaxation time for the sample confirms the quicker ion movements through the samples [53]. Vahini et al. [54] mentioned that small relaxation time led to high ionic conductivity.

Rapid ion migration occurs in MC polymer electrolytes. However, the ion aggregations can obstruct the PE’s ionic migration at the 40 wt.% and 50 wt.% NaBr, thus the relaxation time increases, and the conductivity and dielectric constant are decreased due to ion association. The XRD displayed the increase in the amorphous phase in the MC:NaBr films, while some peaks with high intensity appeared in the XRD pattern of MCNAB4 and MCNAB55 samples associated with the undissolved salts in these samples. The XRD and EIS results agree as the conductivity decreased for the MCNAB4 and MCNAB5 films.

### 3.4. Electric Modulus Study

**Figure 10:**
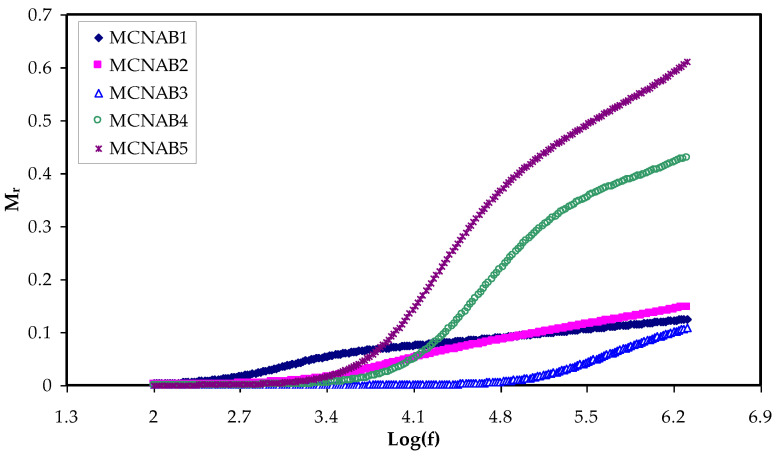
*M_r_* against frequency at room temperature for each sample.

**Figure 11:**
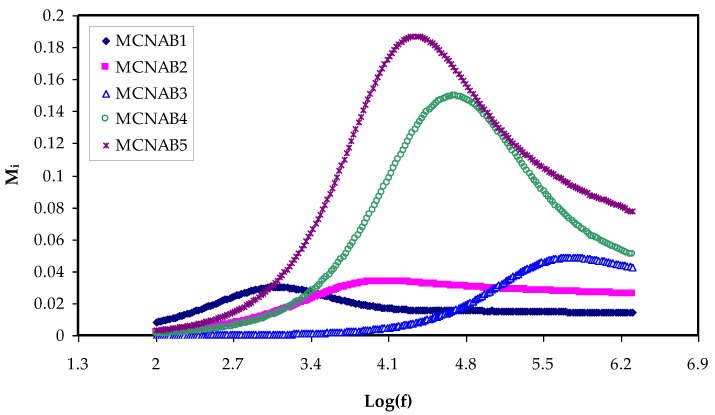
*M_i_* against frequency at room temperature for each sample.

The establishment of a relationship between ionic conductivity and relaxation time during analysis is represented by M* [55]. Interestingly, the relaxation phenomena examination by the electric modulus is superior to conductivity relaxationand permittivity treatments. This is due to suppressing the high value of *ɛ_r_* and *ɛ_i_* at the low frequency. Additionally, the difficulties in dielectric spectrum analysis can be fixed by neglecting the injection of absorbed impurityand space charge [56]. Figure 10 and Figure 11 show that the *M_r_* and *M_i_* values approach zero due to the large capacitance of the double-layer charges at the low-frequency region [16,57]. When the dielectric constant and *M_r_* spectra are compared, it is clear that the *M_r_* are completely different. The high value is recorded for dielectric constant at low frequency, as exhibited in Figure 5.

In contrast, a minimum value is recorded for electric modules (*M_r_* and *M_i_*) at the high frequency, as seen in Figure 10 and Figure 11. This is because the complex electric modules are the reciprocal of the complex dielectric constant. Figure 11 clearly reveals the imaginary part of M^*^ where the peak of the conductivity relaxation is observed. It is perceived that increasing NaBr cause shifting the relaxation peak to low frequency side, indicating an increase in relaxation time (*τ_o_* = 1/*ω_max_*). This means decreasing of segmental mobility in the amorphous phase of the samples with increasing relaxation time. In Figure 11, the responses (peaks) are caused by short-range polymer segmental movement (dipolar mobility) and translational long-range ion movement (translational mobility). In other words, the charges are restricted to potential wells and moving within a short distance at the high frequency range [58,59]. In the low frequency region, the peaks result from the dipole molecules reorienting at sufficient time with the polarization and alternating EF. Overall, all these occurrences produce a double capacitance layer between the electrode and electrolyte interface. Thus, increasing dielectric constant values and relatively very low *M_i_* values are recorded. On the other hand, the stimulus caused by the ions can only perform local (re-orientation) motion results in responses in the form of peaks at the high frequency side [60,61]. There is presence and absence of peaks in *M_i_* spectra and in *ɛ_i_*, respectively (see Figure 6 and Figure 11). Absence or occurrence of peaks in frequency against imaginary EIS plot is related to the effect of space charge and non-localized conductivity. *M″* plots are beneficial in comprehending the behavior of the system when the relaxation processes are speculated to happen owing to ion movement and the electrode impacts are suppressed [47,62]. One more interesting observation is the asymmetric peak maxima in the *M_i_* plot that will not be predicted by ideal Debye behavior [63]. In comparison, the *ε_i_* is larger than the *ε_r_* values at the low frequencies as a result of the existence of free charge motion within the materials [64]. It has also been established that dielectric relaxation is primarily interpreted from the dipole reorientation processes of the polymer chains by a peak appearing in *ε_i_* spectra. However, the ionic motion cooperatively leads to the relaxation peak not appearing in the *ε_i_* diagram [65].

**Figure 12:**
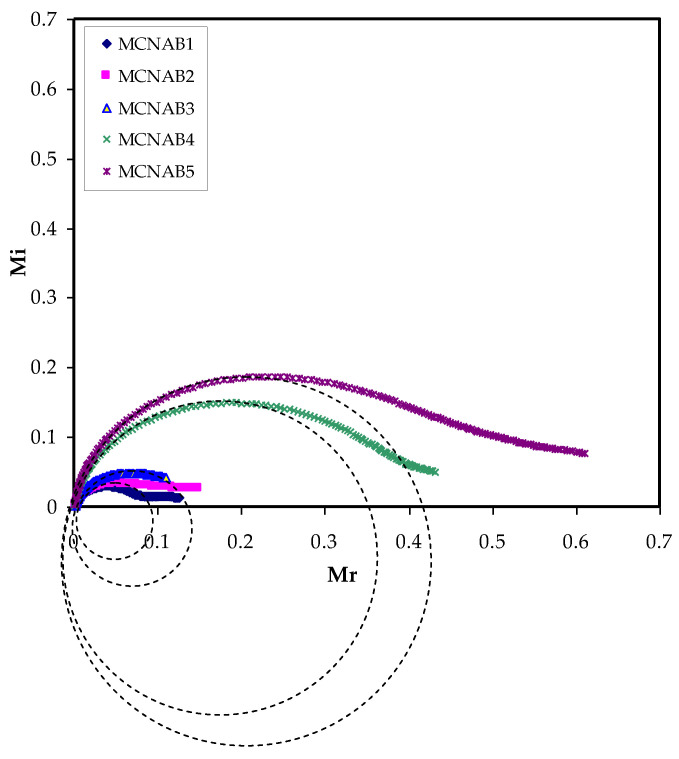
Argand plot at room temperature for each electrolyte.

Additionally, the *M_i_* against *M_r_* plot, which is called Argand plot for all the films, is shown in Figure 12. The Argand plot indicates an incomplete semicircle and it is extrapolated to suit all the film curves. This distorted semicircle is usually an indicator for the wide relaxation times in the systems and shows the non-Debye behavior [66]. The conductivity of each electrolyte is determined to be directly affected with the arc radius in the Argand plot in which the smallest arc contributes to a larger value of conductivity [67]. This relation is also associated with the electrolyte resistivity. This is related to Equation (10), as the maximum conducting electrolyte (MCNAB 3) curve is nearer to the origin [18].

## 4. Conclusions

In this work, polymer electrolytes of MC doped with NaBr were synthesized using the solution cast technique. FTIR and XRD approaches were used to categorize the structural changes in solid films. At 40 and 50 wt.% of NaBr, there were sharp crystalline peaks due to protruded salt distinguished at the XRD pattern. The EIS indicated a decrease in bulk resistance up to MCNAB3 sample (30 wt.% NaBr), which shows an increase of carriers. Beyond this concentration, bulk resistance starts increasing. Using EIS, the loading of NaBr increased the conductivity of the electrolytes. The MCNAB 3 sample obtained a conductivity of 6.71 × 10^−6^ S/cm. To acquire extra understanding of the electrical property of the electrolyte films, the EIS data were fitted by the electrical equivalent circuit (EEC). This behavior model was further confirmed by the incomplete semicircle arc presence in the Argand plot. The conductivity of each film is related to its dielectric property. At low frequency, the large dielectric constant value was seen due to electrode polarization. From electric modulus and loss tangent, the extensive nature of the peaks emerged in the tanδ and electric modulus imaginary parts, showing the distribution of relaxation times. The relaxation time distribution confirms the non-Debye behavior of the electrolytes.

## Figures and Tables

**Table 1:** The elements of SPE films.

Designation	NaBr (wt.%)	MC (g)	NaBr (g)
MCNAB1	10	1.00	0.1111
MCNAB 2	20	1.00	0.2500
MCNAB 3	30	1.00	0.4286
MCNAB 4	40	1.00	0.6666
MCNAB 5	50	1.00	1.0000

**Table 2:** The parameters of the circuit elements of the films at room temperature.

Sample	n1 (Rad)	n2 (Rad)	Y1 (F)	Y2 (F)	R_b_ (Ohm)	Conductivity (S/cm)
MCNAB 1	0.84		1.59 × 10^−9^		8.00 × 10^5^	1.93 × 10^−8^
MCNAB 2	0.90	0.50	1.25 × 10^−9^	2.56 × 10^−7^	8.00 × 10^4^	1.93 × 10^−7^
MCNAB 3	0.83	0.54	3.33 × 10^−9^	1.43 × 10^−6^	2.30 × 10^3^	6.71 × 10^−6^
MCNAB 4	0.83	0.67	4.00 × 10^−10^	4.00 × 10^−7^	9.50 × 10^4^	1.62 × 10^−7^
MCNAB 5	0.85	0.73	3.33 × 10^−10^	1.37 × 10^−7^	2.48 × 10^5^	6.22 × 10^−8^

## Data Availability

Not applicable.

## References

[1] Chen Z., Pei J., Li R. (2017). Study of the Preparation and Dielectric Property of PP/SMA/PVDF Blend Material. Appl. Sci..

[2] Di Noto V., Negro E., Lavina S., Vittadello M. (2010). Hybrid inorganic–organic polymer electrolytes. Polymer Electrolytes.

[3] Asnawi A., Hamsan M., Aziz S., Kadir M., Matmin J., Yusof Y. (2021). Impregnation of [Emim] Br ionic liquid as plasticizer in biopolymer electrolytes for EDLC application. Electrochim. Acta.

[4] Aziz S., Dannoun E., Hamsan M., Abdulwahid R., Mishra K., Nofal M., Kadir M. (2021). Improving EDLC Device Performance Constructed from Plasticized Magnesium Ion Conducting Chitosan Based Polymer Electrolytes via Metal Complex Dispersion. Membranes.

[5] Aziz S., Nofal M., Kadir M., Dannoun E., Brza M., Hadi J., Abdullah R. (2021). Bio-Based Plasticized PVA Based Polymer Blend Electrolytes for Energy Storage EDLC Devices: Ion Transport Parameters and Electrochemical Properties. Materials.

[6] Aziz S., Asnawi A., Kadir M., Alshehri S., Ahamad T., Yusof Y., Hadi J. (2021). Structural, Electrical and Electrochemical Properties of Glycerolized Biopolymers Based on Chitosan (CS): Methylcellulose (MC) for Energy Storage Application. Polymers.

[7] Ngai K.S., Ramesh S., Ramesh K., Juan J.C. (2016). A review of polymer electrolytes: Fundamental, approaches and applications. Ionics.

[8] Aziz S.B. (2013). Li+ ion conduction mechanism in poly (ε-caprolactone)-based polymer electrolyte. Iran. Polym. J..

[9] Aziz S.B., Brza M.A., Nofal M.M., Abdulwahid R.T., Hussen S.A., Hussein A.M., Karim W.O. (2020). A Comprehensive Review on Optical Properties of Polymer Electrolytes and Composites. Materials.

[10] Aziz S.B., Abidin Z.H.Z. (2014). Electrical and morphological analysis of chitosan: AgTf solid electrolyte. Mater. Chem. Phys..

[11] Agrawal R.C., Pandey G.P. (2008). Solid polymer electrolytes: Materials designing and all-solid-state battery applications: An overview. J. Phys. D Appl. Phys..

[12] Brza M.A., Aziz S.B., Nofal M.M., Saeed S.R., Al-Zangana S., Karim W.O., Hussen S.A., Abdulwahid R.T., Kadir M.F.Z. (2020). Drawbacks of Low Lattice Energy Ammonium Salts for Ion-Conducting Polymer Electrolyte Preparation: Structural, Morphological and Electrical Characteristics of CS:PEO:NH_4_BF_4_-Based Polymer Blend Electrolytes. Polymers.

[13] Aziz S.B., Brza M., Saed S.R., Hamsan M.H., Kadir M. (2020). Ion association as a main shortcoming in polymer blend electrolytes based on CS:PS incorporated with various amounts of ammonium tetrafluoroborate. J. Mater. Res. Technol..

[14] Salehan S.S., Nadirah B.N., Saheed M.S.M., Yahya W.Z.N., Shukur M.F. (2021). Conductivity, structural and thermal properties of corn starch-lithium iodide nanocomposite polymer electrolyte incorporated with Al_2_O_3_. J. Polym. Res..

[15] Shetty S.K., Ismayil, Shetty G. (2020). Enhancement of Electrical and Optical Properties of Sodium Bromide Doped Carboxymethyl Cellulose Biopolymer Electrolyte Films. J. Macromol. Sci. Part B.

[16] Agrawal S.L., Singh M., Tripathi M., Dwivedi M.M., Pandey K. (2009). Dielectric relaxation studies on [PEO-SiO_2_]:NH_4_SCN nanocomposite polymer electrolyte films. J. Mater. Sci..

[17] Aziz S.B., Abidin Z.H.Z. (2015). Ion-transport study in nanocomposite solid polymer electrolytes based on chitosan: Electrical and dielectric analysis. J. Appl. Polym. Sci..

[18] Aziz S.B. (2016). Role of Dielectric Constant on Ion Transport: Reformulated Arrhenius Equation. Adv. Mater. Sci. Eng..

[19] Aziz S.B., Abdullah R.M., Rasheed M.A., Ahmed H.M. (2017). Role of Ion Dissociation on DC Conductivity and Silver Nanoparticle Formation in PVA:AgNt Based Polymer Electrolytes: Deep Insights to Ion Transport Mechanism. Polymers.

[20] Pradhan D.K., Choudhary R.N.P., Samantaray B.K. (2008). Studies of Dielectric Relaxation and AC Conductivity Behaviorof Plasticized Polymer Nanocomposite Electrolytes. Int. J. Electrochem. Sci..

[21] Rao V., Ashokan P.V., Shridhar M.H. (2000). Studies of dielectric relaxation and a.c. conductivity in cellulose acetate hydrogen phthalate–poly(methyl methacrylate) blends. Mater. Sci. Eng. A.

[22] Prokhorov E., Luna-Bárcenas G., González-Campos J., Kovalenko Y., García-Carvajal Z., Mota-Morales J. (2016). Proton conductivity and relaxation properties of chitosan-acetate films. Electrochim. Acta.

[23] Reddy C.V.S., Jin A.-P., Zhu Q.-Y., Mai L.-Q., Chen W. (2006). Preparation and characterization of (PVP + NaClO4) electrolytes for battery applications. Eur. Phys. J. E.

[24] Fuentes I., Andrio A., Teixidor F., Viñas C., Compañ V. (2017). Enhanced conductivity of sodium versus lithium salts measured by impedance spectroscopy. Sodium cobaltacarboranes as electrolytes of choice. Phys. Chem. Chem. Phys..

[25] Atkins P. (2010). Shriver and Atkins’ Inorganic Chemistry.

[26] Railanmaa A., Kujala M., Keskinen J., Kololuoma T., Lupo D. (2019). Highly flexible and non-toxic natural polymer gel electrolyte for printed supercapacitors for IoT. Appl. Phys. A.

[27] Aziz S.B., Rasheed M.A., Ahmed H.M. (2017). Synthesis of Polymer Nanocomposites Based on [Methyl Cellulose]_(1−*x*)_:(CuS)_x_ (0.02 M ≤ *x* ≤ 0.08 M) with Desired Optical Band Gaps. Polymers.

[28] Turhan K., Sahbaz F., Güner A. (2001). A Spectrophotometric Study of Hydrogen Bonding in Methylcellulose-based Edible Films Plasticized by Polyethylene Glycol. J. Food Sci..

[29] Aziz N.A.N., Idris N.K., Isa M.I.N. (2010). Solid Polymer Electrolytes Based on Methylcellulose: FT-IR and Ionic Conductivity Studies. Int. J. Polym. Anal. Charact..

[30] Zhu Y., Xiao S., Li M., Chang Z., Wang F., Gao J., Wu Y. (2015). Natural macromolecule based carboxymethyl cellulose as a gel polymer electrolyte with adjustable porosity for lithium ion batteries. J. Power Sources.

[31] Tunç S., Duman O., Polat T.G. (2016). Effects of montmorillonite on properties of methyl cellulose/carvacrol based active antimicrobial nanocomposites. Carbohydr. Polym..

[32] Buslov D.K., Sushko N.I., Tretinnikov O.N. (2008). Study of thermal gelation of methylcellulose in water using FTIR-ATR spectroscopy. J. Appl. Spectrosc..

[33] Liu P., Wei X., Liu Z. (2013). Miscibility Study of Chitosan and Methylcellulose Blends. Adv. Mater. Res..

[34] Salleh N.S., Aziz S.B., Aspanut Z., Kadir M.F.Z. (2016). Electrical impedance and conduction mechanism analysis of biopolymer electrolytes based on methyl cellulose doped with ammonium iodide. Ionics.

[35] Pradhan D.K., Choudhary R.N., Samantaray B.K., Karan N.K., Katiyar R.S. (2007). Effect of Plasticizer on Structural and Electrical Properties of Polymer Nanocompsoite Electrolytes. Int. J. Electrochem. Sci..

[36] Mohapatra S.R., Thakur A.K., Choudhary R.N.P. (2009). Effect of nanoscopic confinement on improvement in ion conduction and stability properties of an intercalated polymer nanocomposite electrolyte for energy storage applications. J. Power Sources.

[37] Aziz S.B., Abdullah R.M. (2018). Crystalline and amorphous phase identification from the tanδ relaxation peaks and impedance plots in polymer blend electrolytes based on [CS: AgNt] x:PEO (x-1)(10 ≤ x ≤ 50). Electrochim. Acta.

[38] Aziz S.B., Abdullah R.M., Kadir M.F.Z., Ahmed H.M. (2019). Non suitability of silver ion conducting polymer electrolytes based on chitosan mediated by barium titanate (BaTiO_3_) for electrochemical device applications. Electrochim. Acta.

[39] Teo L.P., Buraidah M.H., Nor A.F.M., Majid S.R. (2012). Conductivity and dielectric studies of Li_2_SnO_3_. Ionics.

[40] Aziz S.B., Karim W.O., Brza M.A., Abdulwahid R.T., Saeed S.R., Al-Zangana S., Kadir M.F.Z. (2019). Ion Transport Study in CS: POZ Based Polymer Membrane Electrolytes Using Trukhan Model. Int. J. Mol. Sci..

[41] Li C., Huang Y., Feng X., Zhang Z., Gao H., Huang J. (2021). Silica-assisted cross-linked polymer electrolyte membrane with high electrochemical stability for lithium-ion batteries. J. Colloid Interface Sci..

[42] Vani C.V., Thanikaikarasan S., Mahalingam T., Sebastian P., Verea L.E., Shajan X.S. (2014). Effect of X-ray Irradiation on Dielectric Properties of Polymer Electrolytes Complexed with LiCF_3_SO_3_. J. New Mater. Electrochem. Syst..

[43] Tripathi S.K., Gupta A., Kumari M. (2012). Studies on electrical conductivity and dielectric behavior of PVdF–HFP–PMMA–NaI polymer blend electrolyte. Bull. Mater. Sci..

[44] Das S., Ghosh A. (2015). Ionic conductivity and dielectric permittivity of PEO-LiClO_4_ solid polymer electrolyte plasticized with propylene carbonate. AIP Adv..

[45] Aziz S.B., Woo T.J., Kadir M., Ahmed H.M. (2018). A conceptual review on polymer electrolytes and ion transport models. J. Sci. Adv. Mater. Devices.

[46] Aziz S.B. (2015). Study of electrical percolation phenomenon from the dielectric and electric modulus analysis. Bull. Mater. Sci..

[47] Gohel K., Kanchan D. (2018). Ionic conductivity and relaxation studies in PVDF-HFP:PMMA-based gel polymer blend electrolyte with LiClO_4_ salt. J. Adv. Dielectr..

[48] Pawlicka A., Tavares F.C., Dörr D.S., Cholant C.M., Ely F., Santos M.J.L., Avellaneda C.O. (2019). Dielectric behavior and FTIR studies of xanthan gum-based solid polymer electrolytes. Electrochim. Acta.

[49] Marf A.S., Abdullah R.M., Aziz S.B. (2020). Structural, Morphological, Electrical and Electrochemical Properties of PVA: CS-Based Proton-Conducting Polymer Blend Electrolytes. Membranes.

[50] Woo H., Majid S., Arof A. (2012). Dielectric properties and morphology of polymer electrolyte based on poly(ɛ-caprolactone) and ammonium thiocyanate. Mater. Chem. Phys..

[51] Idris N.H., Senin H.B., Arof A.K. (2007). Dielectric spectra of LiTFSI-doped chitosan/PEO blends. Ionics.

[52] Sengwa R., Dhatarwal P. (2020). Predominantly chain segmental relaxation dependent ionic conductivity of multiphase semicrystalline PVDF/PEO/LiClO4 solid polymer electrolytes. Electrochim. Acta.

[53] Ahmed H.T., Jalal V.J., Tahir D.A., Mohamad A.H., Abdullah O.G. (2019). Effect of PEG as a plasticizer on the electrical and optical properties of polymer blend electrolyte MC-CH-LiBF4 based films. Results Phys..

[54] Vahini M., Muthuvinayagam M., Isa M.I.N.M. (2019). Preparation and Characterization of Biopolymer Electrolytes Based on Pectin and NaNO3 for Battery Applications. Polym. Sci. Ser. A.

[55] Pradhan D.K., Choudhary R.N.P., Samantaray B.K. (2008). Studies of structural, thermal and electrical behavior of polymer nanocomposite electrolytes. Express Polym. Lett..

[56] Sengwa R.J., Choudhary S. (2010). Investigation of correlation between dielectric parameters and nanostructures in aqueous solution grown poly(vinyl alcohol)-montmorillonite clay nanocomposites by dielectric relaxation spectroscopy. Express Polym. Lett..

[57] Aziz S.B., Abidin Z.H.Z., Arof A.K. (2010). Influence of silver ion reduction on electrical modulus parameters of solid polymer electrolyte based on chitosan-silver triflate electrolyte membrane. Express Polym. Lett..

[58] Aziz S.B. (2016). Occurrence of electrical percolation threshold and observation of phase transition in chitosan(1−x):AgIx (0.05 ≤ x ≤ 0.2)-based ion-conducting solid polymer composites. Appl. Phys. A.

[59] Castillo J., Chacon M., Castillo R., Vargas R.A., Bueno P.R., Varela J.A. (2009). Dielectric relaxation and dc conductivity on the PVOH-CF_3_COONH_4_ polymer system. Ionics.

[60] Ahmad M.M. (2015). Lithium ionic conduction and relaxation dynamics of spark plasma sintered Li_5_La_3_Ta_2_O_12_ garnet nanoceramics. Nanoscale Res. Lett..

[61] Aziz S.B., Al-Zangana S., Woo H.J., Kadir M.F.Z., Abdullah O.G. (2018). The Compatibility of Chitosan with Divalent Salts over Monovalent Salts for the Preparation of Solid Polymer Electrolytes. Results Phys..

[62] Dave G., Kanchan D.K. (2018). Dielectric relaxation and modulus studies of PEO-PAM blend based sodium salt electrolyte system. Indian J. Pure Appl. Phys..

[63] Aziz S.B., Mamand S.M. (2018). The Study of Dielectric Properties and Conductivity Relaxation of Ion Conducting Chitosan: NaTf Based Solid Electrolyte. Int. J. Electrochem. Sci..

[64] Malathi J., Kumaravadivel M., Brahmanandhan G., Hema M., Baskaran R., Selvasekarapandian S. (2010). Structural, thermal and electrical properties of PVA–LiCF_3_SO_3_ polymer electrolyte. J. Non-Cryst. Solids.

[65] Pradhan D.K., Choudhary R., Samantaray B. (2009). Studies of dielectric and electrical properties of plasticized polymer nanocomposite electrolytes. Mater. Chem. Phys..

[66] Gohel K., Kanchan D.K. (2019). Effect of PC:DEC plasticizers on structural and electrical properties of PVDF–HFP:PMMA based gel polymer electrolyte system. J. Mater. Sci. Mater. Electron..

[67] Sundaramahalingam K., Muthuvinayagam M., Nallamuthu N. (2019). AC Impedance Analysis of Lithium Ion Based PEO:PVP Solid Polymer Blend Electrolytes. Polym. Sci. Ser. A.

